# Arm is the New Leg: A Case Report on Phlegmasia Cerulea Dolens of the Upper Extremity

**DOI:** 10.51894/001c.165680

**Published:** 2026-07-31

**Authors:** Kaye Littell, Michelle Powers

**Affiliations:** 1 Henry Ford - Genesys

## 93

### Introduction

Phlegmasia Cerulea Dolens is a severe form of DVT that is associated with a high morbidity and mortality rate. It is more frequently found in the lower extremities, but can affect upper extremities as well. Without immediate treatment, patients can experience loss of life or limb. It is important to recognize the signs and symptoms, as well as how to diagnose the condition.

### Case Description

A previously healthy 48-year-old female with a remote history of hypothyroidism presented to the emergency department with three days of arm swelling, discoloration, and pain. Her prescription medications included oral contraceptive pill used for heavy menses and family planning. She was evaluated by her primary care provider two days prior for arm pain and swelling; her PCP was concerned for a blood clot and ordered an upper extremity ultrasound with doppler, which was read as negative for DVT. She was then prescribed steroids for the swelling. Her hand became swollen to the point she had to remove her wedding band secondary to pain. On the day she presented to the ER, she stated that she noticed her left arm was becoming discolored. Physical exam showed marked swelling in her left upper extremity, with tense skin noted in the forearm and hand. She had full range of motion, pain that was not exacerbated or relieved by positioning, and notable discoloration with a line of demarcation that was circumferential around her mid upper arm. Bedside POCUS was performed. Deep and superficial veins were all easily compressible in the forearm; in the upper arm, cephalic and basilic veins were compressible. At this point, the patient was positioned supine with her left arm above her head and the basilic vein was tracked proximally to the axillary vein, with the probe at the level of her axilla. There was a visible clot within the vein, which was no longer compressible. Upper extremity doppler ultrasound was ordered and discovered >6cm DVT in the subclavian and axillary veins.

### Discussion

The importance of physical exam, bedside ultrasound, and identifying rare disease processes.

